# A dataset for aqueous surfactant phase behavior as a function of temperature and composition

**DOI:** 10.1038/s41597-025-06306-9

**Published:** 2025-11-26

**Authors:** Felix Rummel, Patrick B. Warren, David J. Bray, Zeynep Sumer, Jonathan Booth, Ardita Shkurti, Richard L. Anderson

**Affiliations:** 1 The Hartree Centre, STFC Daresbury Laboratory, Warrington, WA4 4AD United Kingdom; 2 IBM Research Europe - UK, Warrington, WA4 4AD United Kingdom

**Keywords:** Self-assembly, Cheminformatics

## Abstract

Presented here is a dataset (PhDat) discretizing the aqueous phase behavior of 143 surfactants as a function of temperature and composition. Across the complete dataset, we classify the discretized state points into 118 distinct possible phase states, comprising both single- and two-phase regions, taking a probabilistic approach to describe phase transitions and narrow biphasic gaps. We also outline the workflow adopted to obtain the digitized phase diagrams. We anticipate this dataset will be useful for machine learning or similar applications, in the practically important field of surfactant formulation. The dataset has been designed to be extensible such that it can accommodate a wider variety of surfactant mixtures or non-surfactant molecule phase diagrams.

## Background & Summary

Surfactants are a key ingredient in many formulated products, ranging from pharmaceuticals and cosmetics, to paints and cleaning products^1–8^. Their usefulness stems from their ability to decrease surface tension at interfaces, which allows, for example, immiscible liquids to be blended into emulsions. The action of surfactants can be attributed to their structure, comprising one or more polar or charged ‘head’ groups attached to one or more non-polar ‘tail’ groups^9^. It is convenient to broadly classify surfactants by the state of charge in aqueous solution: thus, ionic surfactants dissociate completely to form surfactant ions and counterions (within this classification, anionic surfactants are negatively charged and cationic surfactants positively charged), nonionic surfactants remain neutral, and zwitterionic surfactants carry both positive and negative charge. In terms of physical chemistry, the phase behavior of surfactants is of particular interest. It is, for example, a key determinant of the rheology, which is relevant both for formulated products, for instance in the home and personal care sector, and also in materials processing.

Thermodynamically, the phase behavior of an aqueous surfactant solution (e.g. a binary surfactant–water mixture) is summarized in a *phase diagram*, which gives the phase state as a function of temperature (and/or pressure) and composition. A full discussion of the rich complexity of surfactant phase behavior is not appropriate for this article, so we limit ourselves to sketching out the main features below. For more details the reader may wish to consult Laughlin’s comprehensive monograph on the subject^1^, or Abbot^10^.

**Fig. 1:**
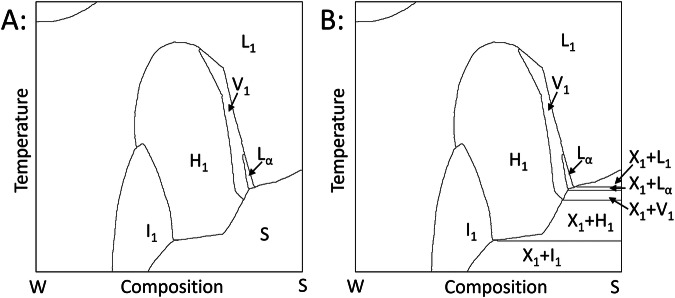
Example of a phase diagram where a broad two-phase region below the freezing point of the surfactant, labelled ‘S’ in the original, is disambiguated by adding horizontal lines as indicated. For the rest of the labelling scheme, see Tables 4 and 5. Reproduced with permission from Ref. ^33^. Copyright 1983 Royal Society of Chemistry.

**Fig. 2:**
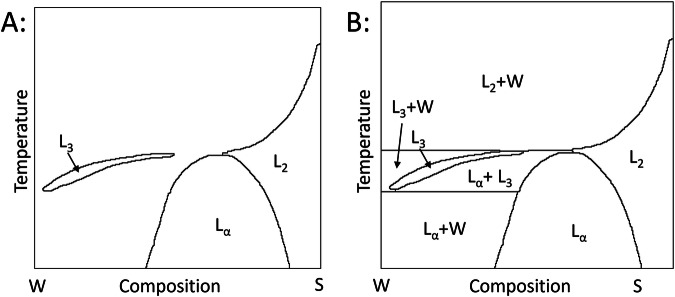
Example of a phase diagram where a large cloud region interrupted by an L_3_ sponge phase (**A**) is augmented for our purposes by adding horizontal lines as indicated (**B**). (**A**) is reproduced from Colloids and Surfaces A: Physicochemical and Engineering Aspects, 84 (1), J.C. Ravey & M.J. Stébé, Properties of fluorinated nonionic surfactant-based systems and comparison with non-fluorinated systems, pg 11-31, Copyright (1984), with permission from Elsevier^34^.

Two prototypical examples of surfactant phase diagrams are shown in Figs. 1a and 2a. At different compositions and conditions the solution exists in different phases or phase states. The phase state depends on how the surfactant self-organizes into assemblies such as micelles, rods, or lamellar sheets. These assemblies themselves may be then further ordered into ‘mesophases’ such as, for example, cubic packings of micelles, hexagonal arrangements of rods, stacked lamellar sheets, and so on.

The properties of these phases vary dramatically, with disordered micellar solutions typically being of low viscosity (unless the micelles grow into rod-like or worm-like structures), and mesophases such as cubic micellar packings, etc, often being highly viscous liquids or even soft solids. Usually the transitions between the mesophases are weakly first-order so that there are narrow biphasic gaps (two-phase regions) between them, and they also often ‘melt’ on increasing temperatures into a disordered liquid phase. Below the freezing point of the surfactant, mesophases or surfactant solutions coexist with various solid surfactant phases, which are often stoichiometrically hydrated crystals. Below the freezing point of water itself, ice appears as an additional phase, leaving a liquid region of diminishing extent which vanishes at a classic eutectic point. Conversely at high temperatures, and low concentrations, many nonionic surfactants exhibit what is usually termed a ‘cloud’ region. This is actually a broad two-phase region where the surfactant solution coexists with essentially excess water. This cloud region may ‘collide’ with the mesophase regions, further enriching the phase diagram and giving rise to yet further phase state possibilities such as a ‘sponge’ phase (dilute, disordered, singly-connected lamellar sheets).

Thus we see that an individual surfactant can exhibit a potentially complicated and rich phase diagram, with many features such as the above-listed intermediate mesophases and two-phase regions. While it is perfectly possible to collect this phase data experimentally, it is highly desirable if one could *predict* the phase behavior, especially for novel surfactants which may not have been synthesized and purified. This is especially attractive in the current surfactant markets, motivated by a pressing move to rapidly decarbonize the multi-billion dollar surfactant supply chain (moving away from petrochemicals and traditional plant-based feedstocks towards sustainably-sourced raw materials).

While simulation approaches such as molecular dynamics and coarse-grained methods such as dissipative particle dynamics are being continuously developed to capture phase behavior and other properties with improving accuracy^11–14^, these methods still possess some shortcomings due to high computational cost and accessible time scales^15,16^. Machine Learning (ML) approaches may offer a dramatically more cost effective alternative to this, potentially enabling the rapid prediction of complete phase diagrams for novel surfactants or filling in partially complete phase diagrams, allowing for a small amount of experimental data to be supplemented by ML data. ML has been used to predict phase diagrams^17–23^ and also other chemical properties based only on relatively simple descriptors such as the SMILES string^24–26^.

All ML approaches rely on the availability of data, with more available data generally resulting in a better outcome^27^. This is also true for simulation approaches which rely upon experimental observables to fit and validate models. To the best of our knowledge, the most complete (although not readily accessible) surfactant phase behavior dataset suitable for use in ML campaigns was collected by Bell^23^. This comprised a dataset for 23 nonionic surfactants covering binary temperature-composition phase diagrams. Bell used this dataset to train ML algorithms and to predict phase diagrams. This was taken further by Thacker and coworkers^17^, one of whose findings was, unsurprisingly, that in order to further improve the predictive power of ML algorithms looking to predict the phase behavior of surfactants, a larger, more comprehensive dataset for surfactant phase behavior must be constructed. This need for more phase diagram data was the motivator behind the work presented in this article. It is hoped that the dataset presented here can be expanded by others in future covering an even wider range of phase diagrams from a more diverse set of surfactants, leading to better simulation and ML models.

Our data discovery effort captured binary aqueous composition/temperature phase diagrams for 143 surfactants found in the literature. These span both nonionic and ionic molecules. A semi-automatic workflow (summarized below) was developed to expedite the data extraction process. Unlike in previous work, where a strict categorical assignment of the phase state was made, in the present dataset the phase state is represented *probabilistically* whereby each composition/temperature point is assigned a probability of being in a given phase state, with single phase regions and two-phase coexistence regions treated on an equal footing. This allows for experimental uncertainties and broadened phase boundaries to be represented more accurately, but is also directly useful for ML approaches.

**Fig. 3:**
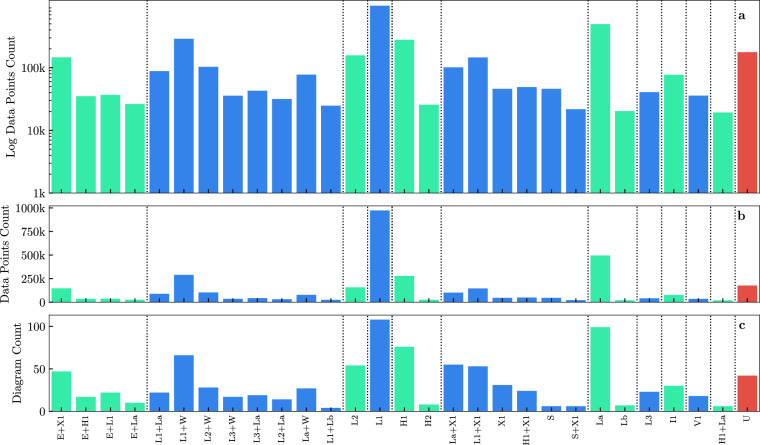
Bar plots indicating how many data points have a non-zero probability for a given phase state. (**a**) showing the log count. (**b**) Showing the count. (**c**) Bar plot indicating how many phase diagrams have a given phase state present. Dashed vertical lines indicate rough groupings of phase state types. For (**a**) and (**b**) a unit grid spacing was used for both temperature and composition axes (i.e. 1 ^°^C and 1 wt% respectively); for the typical phase diagram this usually produced around 10k data points. Here we omit all phases that have a non-zero probability for less than 1% of the total number of data points.

Figure 3 indicates the distribution of data, making up 99 % of the total collected, both in terms of the frequency of occurrence of individual phase states and in terms of how many phase diagrams contain a given phase state. This shows that there is wide spread both in terms of how many diagrams contain a given phase state, and how large a given phase state is in a phase diagram. For example the *L*_1_ phase features as a large area in many phase diagrams, whereas the *L*_*β*_ and *I*_1_ phases are less well represented.

## Methods

**Fig. 4:**
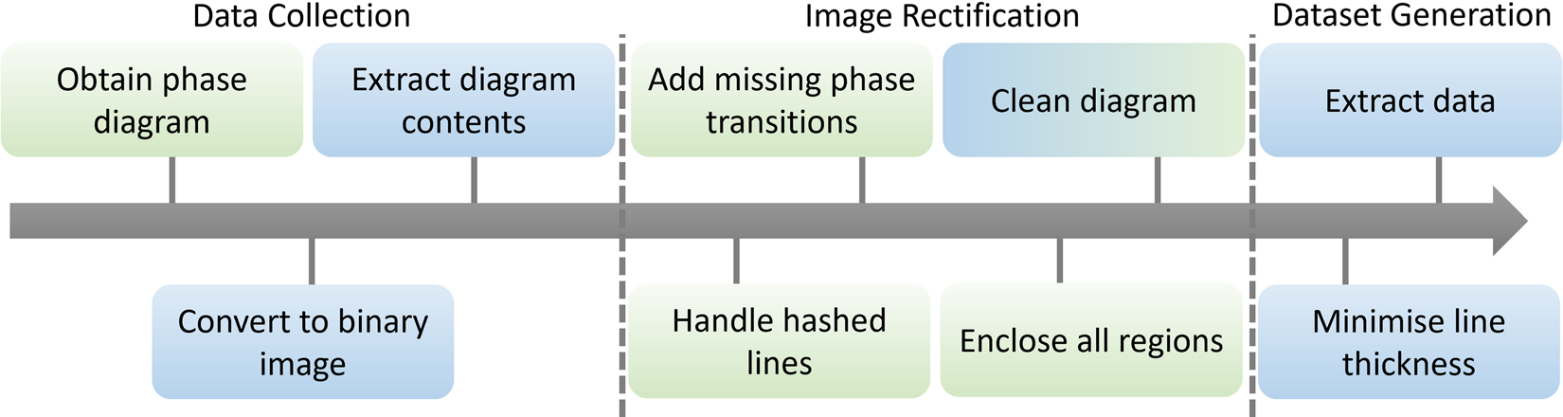
Summary of the workflow for digitizing phase diagrams: blue boxes indicate steps which are readily automated and green boxes represent steps that require manual input; not all diagrams require all the steps.

Previously, Bell discretized the phase behavior manually on a grid of state points and identified the corresponding phase state by visual inspection^23^. While this is a possible method of data collection, though likely resulting in some inherent measurement error, it was desirable to automate this process as far as possible to ease creation of a larger dataset with a chosen grid size for each phase diagram to be collected. With this in mind we automated many steps of the procedure. The final workflow from obtaining the literature data to the final database entry is illustrated in Fig. 4 and outlined below. It can be broken down into three main steps: data collection, image processing, and data extraction.

### Data Collection

Phase diagrams were obtained from a variety of sources, including papers and books as electronic or scanned physical copies. Table 1 summarizes the collected nonionic diagrams, while Table 2 summarizes the ionic diagrams. The surfactant name and SMILES string were determined from the source. If the surfactant was described as polydisperse the average structure was used for naming purposes and the number of chemical groups rounded to the nearest integer using a standardized notation as defined in the Table caption. For example, the compound R_11_COO(EO)_12.8_CH_3_ studied by Fujiwara *et al*.^28^ is renamed as C_11_C(O)E_13_Me in our notation (see Table 1).

**Table 1:** Nonionic surfactants in PhDat detailing database key, surfactant structure, experimental method (assigned an index to save space - see Table 3), reference and figure number.

No.	Structure	Ex.	Rf.	Fg.	No.	Structure	Ex.	Rf.	Fg.
**amine**					**119**	${{\rm{C}}}_{12}{\rm{G}}{({{\rm{E}}}_{4}{\rm{Me}})}_{2}$	A	^35^	2
4^*^	C_10_E_3_CCNMe_2_	F	^1^	5.19	**120**	${{\rm{C}}}_{14}{\rm{G}}{({{\rm{E}}}_{4}{\rm{M}})}_{2}$	A	^35^	2
7^*^	C_8_N	F	^1^	10.19	**121**	${{\rm{C}}}_{16}{\rm{G}}{({{\rm{E}}}_{4}{\rm{M}})}_{2}$	A	^35^	2
**ester**					**122**	${{\rm{C}}}_{14}{\rm{G}}{({{\rm{E}}}_{3}{\rm{M}})}_{2}$	A	^35^	2
**14**	C_8_C=CC_7_C (=O) G	B	^36^	2	**123**	${{\rm{C}}}_{14}{\rm{G}}{({{\rm{E}}}_{5}{\rm{M}})}_{2}$	A	^35^	2
**15**	C_4_C=CC_7_C (=O) G	B	^37^	4	**miscellaneous**				
**16**	C_4_C=CC8C (=O) G	B	^38^	2	8^*^	C_11_C (=O) NMeC(CO)_5_	F	^1^	10.21
**31**^†^	C_11_C (=O) E6Me	A	^28^	2	**38**	C=CC_1_OPh_2_OCC- (=O) E_7_Me	F	^39^	3
**32**^†^	C_11_C (=O) E_5_Me	A	^28^	2	53^*^	C_12_P (=O) (CC*#*N)_2_	F	^1^	5.21
**33**^†^	C_11_C (=O) E_7_Me	A	^28^	2	**124**	(Me)_3_SiC_6_E_5_Me		^40^	7
**34**^†^	C_11_C (=O) E_9_Me	A	^28^	2	84^*^	C_8_FCOO	F	^1^	11.13
**35**^†^	C_11_C (=O) E_13_Me	A	^28^	2	3^*^	C12S(N)_2_Me	F	^1^	5.18
**144**	C_15_C (=O) G	AB	^41^	7	61^*^	C_12_S (=O) Me	F	^1^	10.12
**37**	C_8_C=CC_11_C (=O) G	AB	^42^	2	**75**	C_12_SO_3_	F	^1^	10.6
**117**	C_6_C=CC_9_C (=O) G	B	^43^	2	**perfluoroalkyl ethoxylate**				
**141**	C_5_C (=O) G	AB	^41^	1	**17**	${{\rm{C}}}_{6}^{{\rm{F}}}{{\rm{CE}}}_{4}$	BE	^34^	10
**142**	C_7_C (=O) G	AB	^41^	3	**18**	${{\rm{C}}}_{6}^{{\rm{F}}}{{\rm{CE}}}_{6}$	BE	^34^	11
**143**	C_9_C (=O) G	AB	^41^	4	19^*^	${{\rm{C}}}_{6}^{{\rm{F}}}{{\rm{CE}}}_{5}$	BE	^34^	15
**ethoxylate**					**20**	${{\rm{C}}}_{6}^{{\rm{F}}}{{\rm{CE}}}_{5}{\rm{Me}}$	BE	^34^	15
**36**^†^	C_12_E_13_	A	^28^	4	**21**	${{\rm{C}}}_{7}^{{\rm{F}}}{{\rm{CE}}}_{4}{\rm{Me}}$	BE	^34^	15
5^*^	C_10_E_3_	F	^1^	5.22	**22**	${{\rm{C}}}_{8}^{{\rm{F}}}{\rm{CC}}$ (=O) ${\rm{N}}{({{\rm{E}}}_{3}{\rm{Me}})}_{2}$	BE	^34^	16
9^*^	C_10_PhE_9_	F	^1^	10.23	**23**	${{\rm{C}}}_{10}^{{\rm{F}}}{\rm{CC}}$ (=O) ${\rm{N}}{({{\rm{E}}}_{3}{\rm{Me}})}_{2}$	BE	^34^	19
11^*^	C_10_E_4_G	F	^1^	10.25	**phosphate**				
13^*^	C_10_E_4_	F	^1^	4.5	**64**	C_12_OP (=O) (OMe)_2_	F	^1^	10.13
**39**	C_12_IsoE_3_	A	^44^	4	**65**	C_12_P (=O) (OMe)_2_	F	^1^	10.13
**40**	C_8_E_6_	A	^45^	1a	**67**	C_12_OP (=O) (Me)OMe	F	^1^	10.14
**41**	C_10_E_6_	A	^45^	1b	**phosphine oxide**				
**42**	C_16_E_8_	A	^33^	10	2^*^	C_14_P(Me_2_) = O	F	^46^	6
**43**	C_16_E_12_	A	^33^	11	12^*^	C_10_E_3_CCP(Me_2_) = O	F	^46^	10.25
**44**	C_8_E_4_	A	^33^	3	**54**	C_12_P (=O) Me_2_	F	^46^	10.2
**45**	C12E_3_	A	^33^	4	**62**	C_12_P (=O) Me_2_	F	^46^	10.12
**46**	C_12_E_4_	A	^33^	5	66^*^	C_12_P (=O) ${({{\rm{C}}}_{2})}_{2}$	F	^46^	10.13
**47**	C_12_E_5_	A	^33^	6	70^*^	${{\rm{C}}}_{9}{\rm{C}}({\rm{CO}})\left({\rm{P}}\right.$ (=O) $\left.{{\rm{Me}}}_{2}\right)$	F	^46^	10.2
**48**	C_12_E_6_	A	^33^	7	**72**	C_14_P(Me_2_) = O	F	^46^	10.5
**49**	C_12_E_8_	A	^33^	8	73^*^	C_12_P (=O) ${({{\rm{C}}}_{2})}_{2}$	F	^46^	10.5
**50**	C_16_E_4_	A	^33^	9	74^*^	C_12_P (=O) Me_2_	F	^46^	10.5
**103**	C_12_E_3_	A	^47^	20	86^*^	${{\rm{C}}}_{10}{\rm{P}}{({{\rm{C}}}_{2})}_{2}$=O	F	^46^	11.7
**104**	C_12_E_4_	A	^47^	20	87^*^	${{\rm{C}}}_{8}{\rm{P}}{({{\rm{C}}}_{3})}_{2}$=O	F	^46^	11.7
**105**	C_12_E_5_	A	^47^	20	88^*^	C_6_P(Me)(C_5_)=O	F	^46^	11.7
**112**	C_10_E_6_	A	^48^	3	**89**	${\rm{P}}{({{\rm{C}}}_{5})}_{3}$=O	F	^46^	11.7
**113**	C_10_E_5_	A	^48^	4	**115**	C_10_P (=O) Me_2_	F	^49^	2
**114**	C_10_E_8_	A	^48^	5	**116**	C_12_P (=O) Me_2_	F	^49^	2
**128**	C_12_E_9_	AB	^50^	1	**saccharide**				
**methyl-ethoxylate**					69^*^	C_8_-*β*-D-glucoside	F	^1^	10.20
10^*^	C_10_E_6_Me	F	^1^	10.24	**90**	C_8_-*β*-D-glucopyranoside	BC	^51^	1
**24**	${({{\rm{C}}}_{6})}_{2}{{\rm{GE}}}_{8}{\rm{Me}}$	AB	^52^	2.3	**91**	C_9_-*β*-D-glucopyranoside	BC	^51^	f1
**25**	${({{\rm{C}}}_{7})}_{2}{{\rm{GE}}}_{8}{\rm{Me}}$	AB	^52^	2.3	92^*^	C_10_-*β*-D-glucopyranoside	BC	^51^	1
**26**	${({{\rm{C}}}_{8})}_{2}{{\rm{GE}}}_{8}{\rm{Me}}$	AB	^52^	2.3	125^*^	C_8_-glucoside	BC	^53^	3
**27**	${({{\rm{C}}}_{7})}_{2}{{\rm{GE}}}_{10}{\rm{Me}}$	AB	^52^	2.3	**sulfoximine**				
**28**	${({{\rm{C}}}_{7})}_{2}{{\rm{GE}}}_{6}Me$	AB	^52^	2.3	1^*^	C_12_S (=O) (N)Me	F	^46^	
**29**	${{\rm{C}}}_{12}{\rm{G}}{({{\rm{E}}}_{4}{\rm{Me}})}_{2}$	AB	^52^	7	**6**	C_8_S (=O) (N)Me	F	^1^	
30^*^	C_12_E_8_Me	F	^52^	7					

In the structures, *n* is the alkyl chain length. Me, E_m_, G and Ph are methyl (CH_3_), ethoxy (${[{{\rm{O}}{\rm{C}}{\rm{H}}}_{2}{{\rm{C}}{\rm{H}}}_{2}]}_{{\rm{m}}}{\rm{O}}$) where *m* refers to the number of repeat units, triglyceryl/glycerol unit and phenyl ring groups respectively. Iso is isosorbide ring. Incomplete diagrams are denoted with an asterisk. Where an average ethoxy length was reported E is given to the nearest integer (indicated by a dagger).

**Table 2:** Ionic surfactants in PhDat detailing database key, surfactant structure, experimental method in original reference (assigned an index to save space - see Table 3), reference and figure number.

No.	Structure	Ex.	Rf.	Fig.	No.	Structure	Ex.	Rf.	Fig.
**anion**					**108**	C_12_N^+^(Me)_2_. Cl^−^	A	^47^	22
71^*^	${{\rm{C}}}_{8}{\rm{C}}{({{\rm{C}}}_{4})}_{2}{{\rm{CO}}}_{2}^{-}{{\rm{Na}}}^{+}$	F	^1^	10.4	**109**	(C_12_)(Me) − pip. Br^−^	AB	^47^	5
**76**	${{\rm{Hx}}}_{\beta }^{{\rm{B}}}{\rm{C}}({{\rm{CO}}}_{2}){{\rm{SO}}}_{2}^{-}{{\rm{Na}}}^{+}$	F	^1^	10.7	**110**	(C_14_)(Me) − pip. Br^−^	AB	^47^	5
77^*^	${{\rm{Hx}}}_{\beta }^{{\rm{B}}}{\rm{C}}({{\rm{CO}}}_{2}^{-}){{\rm{SO}}}_{2}^{-}{{\rm{Mg}}}^{2+}$	F	^1^	10.7	**137**	C_12_N^+^(Me)_3_. Cl^−^	F	^54^	6
**78**	${{\rm{Hx}}}_{\beta }^{{\rm{B}}}{\rm{C}}({{\rm{CO}}}_{2}^{-}){{\rm{SO}}}_{2}^{-}{{\rm{Ca}}}^{2+}$	F	^1^	10.7	**111**	(C_16_)(Me) − pip. Br^−^	AB	^47^	5
79^*^	${{\rm{Hx}}}_{\beta }^{{\rm{B}}}{\rm{C}}({{\rm{CO}}}_{2}^{-}){{\rm{SO}}}_{2}^{-}{{\rm{Ba}}}^{2+}$	F	^1^	10.7	**138**	C_16_N^+^(Me)_3_. Br^−^	F	^54^	6
85^*^	${{\rm{CO}}}_{2}^{-}{{\rm{Na}}}^{+}$	F	^1^	11.6	**140**	${{\rm{N}}}^{+}{({\rm{Me}})}_{2}{({{\rm{C}}}_{12})}_{2}.{{\rm{Br}}}^{-}$	F	^54^	8
**93**	${{\rm{C}}}_{11}{{\rm{CO}}}_{2}^{-}{{\rm{K}}}^{+}$	A	^55^	4	**mixed**				
**94**	C_8_C=${{\rm{CC}}}_{7}{{\rm{CO}}}_{2}^{-}{{\rm{K}}}^{+}$	A	^55^	5	80^*^	${{\rm{N}}}^{+}{({{\rm{C}}}_{4})}_{4}.{{\rm{C}}}_{11}{{\rm{CO}}}_{2}^{-}$	F	^1^	10.8
**95**	C_13_CO_2_ − K^+^	A	^55^	6	81^*^	${{\rm{N}}}^{+}{{\rm{Me}}}_{4}.{{\rm{C}}}_{11}{{\rm{CO}}}_{2}^{-}$	F	^1^	10.8
**96**	C_15_CO_2_ − K^+^	A	^55^	7	82^*^	$2({{\rm{MePhSO}}}_{3}^{-}).{{\rm{C}}}_{12}{{\rm{N}}}^{+}$ (Me)_2_C_2_E_7_C_2_N^+^ (Me)_2_C_12_	F	^1^	10.9
**97**	C_17_CO_2_ − K^+^	A	^55^	8	**129**	${{\rm{C}}}_{11}{{\rm{CO}}}_{2}^{-}.{{\rm{C}}}_{12}{{\rm{N}}}^{+}$	F	^54^	10
**99**	${{\rm{C}}}_{12}{{\rm{SO}}}_{4}^{-}{{\rm{Na}}}^{+}$	F	^1^	5.4	**130**	${{\rm{C}}}_{11}{{\rm{SO}}}_{4}^{-}.{{\rm{C}}}_{12}{{\rm{N}}}^{+}$	F	^54^	10
100^*^	${{\rm{C}}}_{15}{{\rm{CO}}}_{2}^{-}{{\rm{Na}}}^{+}$	F	^1^	3.2	**131**	${{\rm{C}}}_{11}{{\rm{SO}}}_{4}^{-}.{{\rm{C}}}_{12}{{\rm{N}}}^{+}{({\rm{Me}})}_{3}$	F	^54^	10
101^*^	${{\rm{C}}}_{11}{{\rm{CO}}}_{2}^{-}{{\rm{Na}}}^{+}$	A	^56^	1	**132**	${{\rm{C}}}_{11}{{\rm{SO}}}_{4}^{-}.{{\rm{C}}}_{12}{{\rm{N}}}^{+}{({\rm{Me}})}_{2}{{\rm{C}}}_{2}$	F	^54^	10
**102**	C_8_C=${{\rm{CC}}}_{7}{{\rm{CO}}}_{2}^{-}{{\rm{Na}}}^{+}$	A	^57^	17	**133**	${{\rm{C}}}_{8}{{\rm{SO}}}_{3}^{-}.{{\rm{C}}}_{7}$-pyridinium	F	^54^	12
**106**	${{\rm{C}}}_{11}{{\rm{CO}}}_{2}^{-}{{\rm{Na}}}^{+}$	A	^47^	21	**134**	${{\rm{C}}}_{8}{{\rm{SO}}}_{3}^{-}.{{\rm{C}}}_{8}$-pyridinium	F	^54^	12
**126**	C_11_C (=O) ${\rm{N}}({{\rm{C}}}_{2}{{\rm{CO}}}_{2}^{-})$ ${{\rm{C}}}_{2}{\rm{N}}({{\rm{C}}}_{2}{{\rm{CO}}}_{2}^{-}){\rm{C}}$ (=O) C_11_. 2Na^+^	AB	^58^	2	**zwitterion**				
**127**	C_11_C (=O) N(Me)C_2_ ${{\rm{CO}}}_{2}^{-}{{\rm{Na}}}^{+}$	AB	^58^	2	51^*^	${{\rm{C}}}_{10}{{\rm{N}}}^{+}{({\rm{Me}})}_{2}{{\rm{C}}}_{3}{{\rm{OSO}}}_{3}^{-}$	F	^1^	5.20
135^*^	Sodium taurodeoxycholate	F	^54^	13	56^*^	${{\rm{C}}}_{16}{{\rm{N}}}^{+}{({\rm{Me}})}_{2}{{\rm{C}}}_{3}{{\rm{SO}}}_{3}^{-}$	F	^1^	10.10
**139**	C_4_C(C_2_)COC (=O) CC $({{\rm{SO}}}_{3}^{-}){\rm{C}}$ (=O) OCC (C_2_)C_4_	F	^54^	8	57^*^	C_14_N^+^(Me)_2_C_3_*SO*3^−^	F	^1^	10.10
**cation**					58^*^	${{\rm{C}}}_{12}{{\rm{N}}}^{+}{({\rm{Me}})}_{2}{{\rm{C}}}_{3}{{\rm{SO}}}_{3}^{-}$	F	^1^	10.10
**52**	C_12_N^+^Cl^−^	F	^1^	5.11	59^*^	${{\rm{C}}}_{2}2{{\rm{N}}}^{+}{({\rm{Me}})}_{2}{{\rm{E}}}_{8}{{\rm{C}}}_{2}{{\rm{SO}}}_{3}^{-}$	F	^1^	10.11
**55**	${{\rm{N}}}^{+}{({{\rm{C}}}_{18})}_{2}{({\rm{Me}})}_{2}{{\rm{Cl}}}^{-}$	F	^1^	2.2	60^*^	${{\rm{C}}}_{2}2{{\rm{N}}}^{+}{({\rm{Me}})}_{2}{{\rm{E}}}_{8}{{\rm{C}}}_{2}{{\rm{SO}}}_{4}^{-}$	F	^1^	10.11
**68**	C_12_N^+^(Me)_2_CC (=O) N	F	^1^	10.17	63^*^	C_12_N^+^(Me)_2_O^−^	F	^1^	10.12
83^*^	2Cl^−^. C_12_N^+^(Me)C_2_E_7_ C_2_N^+^(Me)_2_C_12_	F	^1^	10.9	**118**	C_12_N^+^(Me)_2_O^−^	DE	^59^	8
**107**	C_12_N^+^Me. Cl^−^	A	^47^	22	**136**	DPPC	F	^54^	18

In the structures, *n* is the alkyl chain length, Me, E_m_, ${{\rm{Hx}}}_{\beta }^{{\rm{B}}}$, pip, DPPC and Ph are methyl (CH_3_), ethoxy (${[{{\rm{O}}{\rm{C}}{\rm{H}}}_{2}{{\rm{C}}{\rm{H}}}_{2}]}_{{\rm{m}}}{\rm{O}}$) where *m* refers to the number of repeat units, branched hexanol (OCC^*β*^CC(C)CC, attached at the *β* carbon), piperidinium, dipalmitoyl- phosphatidylcholine and phenyl ring groups respectively. Entries with an asterisk are incomplete diagrams.

**Table 3:** Mapping between index given for experimental method in Tables 1 and 2 and the measurement type employed in the original literature.

Index	Description
A	Polarized optical microscopy
B	X-ray
C	Proton nuclear magnetic resonance spectroscopy
D	Calorimetry
E	Other
F	Not available

Initially the selection was sorted by visual inspection into 99 complete diagrams and 44 incomplete diagrams. If phases were ambiguous in their definition or boundaries and it was unclear on where the phase transitions are then the diagram was classed as incomplete otherwise it was classed as complete. Further, for this work only binary (water/surfactant) phase diagrams with numerically labeled temperature and composition axes were retained.

#### Assignment of Phase State Labels

A total of 118 unique phase states (both one- and two-phase regions) were identified, with the additional symbol U being used as a label for unknown regions in incomplete diagrams. The single phase regions are described in Table 4 and a comprehensive list of all phase states (i.e. both one- and two-phase regions) is given in Table 5. To manage this a consistent naming scheme was substituted over the original labels given in the diagrams. This often required detailed perusal of the source text in addition to the phase diagram itself, and some previous familiarity with surfactant phase science was invaluable.

**Table 4:** Adopted phase state labels for single phase regions, across all mapped phase diagrams.

Dataset Label	Lit. Label	Description
E	Ice	Ice
H1	*H*_1_	Hexagonal phase
H2	*H*_2_	Inverse hexagonal phase
I1	*I*_1_	Cubic micellar phase
I2	*I*_2_	Inverse cubic micellar phase
L1	*L*_1_	Isotropic micellar solution
L2	*L*_2_	Isotropic reverse micellar solution
L3	*L*_3_	Sponge phase
La	L_*α*_	Lamellar phase
La1	Soap Boilers Neat Soap	Lamellar phase
La2	Superneat Soap	Lamellar phase
Lb	L_*β*_	Gel phase
M1	M_*α*_	2D Monoclinic phase
N1	*N*_1_	Nematic liquid of rod- or worm-like micelles
Pb	P_*β*_’	Hydrated bilayer-based rippled phase
R1	R_*α*_	Rhombohedral phase
S	Neat Soap	Non-crystalline solid
Sa	S_*α*_	Non-crystalline solid
Sb	S_*β*_	Non-crystalline solid
T1	T_*α*_	Tetragonal phase
V1	*V*_1_	Bicontinuous cubic phase
V2	*V*_2_	Inverse bicontinuous phase
V2i	*V*2_*i*_	Inverse bicontinuous phase with space group Pn3m
V2m	*V*2_*m*_	Inverse bicontinuous phase with space group Ia3d
V2p	*V*2_*p*_	Inverse bicontinuous phase with space group Im3m
W	W	Water phase or sub-micellar solution
X	*X*	Generic crystalline (solid) surfactant phase
X1, X2	*X*_1_, *X*_2_	Solid surfactant phases (differing by hydration state)
U		Unmeasured / unknown / unclear region or phase state

Presented are both the phase state label used in the data set and the corresponding typical literature label. To allow for future expansion we include an I2 cubic micellar and V2m phase although it is not needed in the present dataset. The X1 and X2 labels distinguish solid surfactant phases with different hydration states and are needed in phase diagrams where there is X1+X2 coexistence.

**Table 5:** List of all 118 identified phase states (excluding U) across all sources, adopting the labeling scheme in Table 4.

E+H1	E+I1	E+L1	E+L2	E+La	E+V1	E+X	E+X1	E+X2	E+X3
H1	H1+V2	H1+L1	H1+La	H1+La1	H1+R1	H1+V1	H1+X2	H1+X1	H1+I1
H1+X4									
H2	H2+L1	H2+X1	H2+W	H2+V2p					
I1	I1+X1	I1+X4	I1+X5	I1+T1	I1+La				
I2									
L1	L1+L3	L1+La	L1+La1	L1+Lb	L1+V1	L1+W	L1+X	L1+X1	L1+X2
L1+X3	L1+X4	L1+X6	L1+Sa						
L2	L2+La	L2+Lb	L2+W	L2+X1	L2+Xa	L2+V2i			
L3	L3+La	L3+W	L3+X1						
La	La1	La2	La1+La2	La+S	La+Sa	La+Sb	La+T1	La+V2i	La+V2p
La+W	La+X	La+Xb	La+X1	La+X2	La+X3	La+X4			
Lb	Lb+Pb	Lb+W							
N1									
M1	M1+X1								
Pb	Pb+W								
R1	R1+X1								
S	Sa	Sb	Sa+Sb	S+X1					
T1	T1+X1								
V1	V1+X1								
V2	V2i	V2p	V2i+V2p	V2+W	V2p+W	V2+X1	V2i+X1	V2p+X1	V2i
V2p+X1	V2i+V2p	V2p	V2p+W						
W+X1	W+X2								
X	X1	X1+X2	X2	X2+X3	X3	X3+X4	X4	X5	X6
Xa	Xb								

Two-phase coexistence regions are indicated by a ‘+’ between the corresponding phases, with the phase labels ordered alphabetically for convenience (e.g. W+X1 rather than X1+W). Not all combinations are encountered since the two-phase regions must occur between single-phase regions, and in any given phase diagram the phase sequence is strictly ordered.

Having digitized a wide array of experimentally collected phase diagrams it became clear that there are large differences between sources in the way phase diagrams are presented. Some of this may well be due to some diagrams dating back several decades but also more modern papers still show differences. These include not just labeling variations such as a lamellar phase being reported either as *D*, *L*_*α*_ or *G*, but also the indication of phase boundaries, uncertainty and disputed, unknown or unidentified phases in older diagrams etc.

For the future, we recommend that when reporting phase diagrams a clear description of the labels should be provided. Further, each region on the phase diagram should be labeled, describing an unlabeled region in the text only creates extra work when extracting the diagrams. Additionally it is generally clearer to have diagrams without grid lines, or have them in a different color or thickness, the same goes for indicating tie lines in two-phase regions. Further it is not always clear if some regions are simply broad two-phase regions or phase transitions, especially when these are not labeled, as such it may be beneficial to indicate transitions by solid lines and simply change their thickness. It may also be helpful to publish the raw data along with the phase diagram to allow for direct use of this.

#### Diagram Digitization

Since the phase diagrams were obtained from a variety of sources, it was necessary to design and establish a consistent methodology to extract a digital image of the phase diagram from various media. Screen capturing or figure downloads, where available, were used for electronic phase diagrams, whilst a scan of the phase diagram was used as a starting point for physical papers and books. Using a custom collection of Python scripts packaged into a user interface named CurveClaw^29^ these images were processed. The workflow required the diagram to be fully enclosed on all four sides. While most diagrams were already presented as such, in some cases it was necessary to do this manually using image editing software such as Paint. In these cases lines visually parallel to the axis of the plot were added to enclose the diagram. Each image is then loaded and converted it to binary format. The largest contour by area is then used to identify the graph area. The contents of the contour are then used to extract the phase diagram from the original binary image. In cases where the diagram did not have straight axis, but was distorted, often the case with scans taken from books, the four corner points were translated to a rectangle. As a result a cropped image, containing only the contents enclosed by the outline of the diagram, was retained for further use.

For all phase diagrams sampled, the temperature and composition values were directly extracted from the corresponding original publications, as listed in Tables 1 and 2. Each phase diagram was digitized to preserve the full numerical range reported in the source literature, typically spanning 0–100 ^°^C (min. −55 ^°^C, max. 420 ^°^C) and 0–100 wt% surfactant. Full temperature and composition ranges can be found in the published dataset. No extrapolation or interpolation beyond the published data was performed except as described in this article.

### Image Rectifying

To proceed to data extraction, each phase shown on the image needs to be well defined, such that the phase domain is fully enclosed by solid boundaries. To do this, the following rectifications where applied to the image as required. If a phase transition was defined by both a dashed-line and a solid-line running in parallel close to each other the dashed-line was removed. If a phase transition was only defined by a dashed-line the line was made solid. If a phase transition was defined by two parallel dashed-lines (or solid-lines) a solid-line was drawn in the middle and both dashed-lines removed. If an area was left open the boundary line was continued at its current slope until it intercepted the edge of the diagram or another boundary line. Some diagrams indicated more gradual phase transitions by adding additional dashed or solid lines either horizontally across the diagram or in parallel to a phase transitions, these lines were removed. All labels, arrows, data points and other marks not indicating a phase transition were removed. Below the freezing point of the surfactant, horizontal lines (i.e. isotherms) were added to delineate between two-phase regions with different coexisting phases. Eutectic and peritectic points (both cases where three phases coexist at a single temperature) were also identified and the appropriate horizontal lines drawn in. Examples of how phase diagrams were edited in this way are shown in Figs. 1 and 2 respectively. Finally, each extracted diagram was inspected manually to ensure all phase transitions of the original phase diagram were captured correctly throughout the process.

#### Image of unique phases

Once the image was rectified, the boundary lines were thinned to a minimum, by sequentially removing pixels in order of their adjacency to white pixels without increasing the number of areas present in the image. This maximized the image area that could be sampled effectively (i.e. well defined as a specific phase) and ensured all phase transition lines were of equal width (namely one pixel). This produces a binary image where ‘1’ (i.e. black) represents part of a domain’s boundary line and ‘0’ (i.e. white) the middle of the phase domain. The image can next be converted to identify unique phase domains by labeling phase states by using scipy.ndimage.label() in Python, using the cleaned binary image as input; conveniently the number of phases *n* in the image is then simply the maximum pixel value in the output of this function. The value of *n* (≤118) will vary between phase diagrams but the numerical value cannot be simply substituted for the actual phase state label; rather a curated, unique, individualised mapping is required for each image to convert the numbered regions to the standardized phase state labels.

### Dataset Generation

To create a dataset from the image, a grid of sample points was extracted from the image using the range of composition and temperature of the phase diagram and specifying the grid resolution. Here a common resolution of 1 ^°^C and 1 wt% was used (although the user may specify any desired grid spacing). Hence, each grid point was mapped to the equivalent test pixel on the image (rounding to the nearest pixel as required). Next, for each of these test pixels, the distance to all the pixels in a given phase was calculated. Since each pixel is simply a value in an array the row and column difference between two pixels can be used to obtain the distance they are apart on each axis. Here we treat the 1 ^°^C and 1 wt% steps to be of equal length to obtain the final Euclidean distance between any two points. However different weightings could be used also. The minimum distance, *d*_*i*_, of the test pixel to each phase *i*: *i* = 1, *n* is used to assign the phase state probability, *P*_*i*_, of the test pixel according to ${P}_{i}={e}^{-{d}_{i}/2}$. Note that if a test pixel is in the same phase the distance to that phase is zero and as such *P*_*i*_ = 1, while if test pixel falls onto a boundary pixel it will be equally likely to be in adjacent phases. Finally, for each test pixel, all probabilities below the threshold of 10^−3^ were set to zero for simplicity and the resulting probabilities were normalized to one. This resulted in the final output matrix, with each row corresponding to a particular temperature and composition and each column corresponding to the probability of being in a particular phase state.

### Automation of Image cleaning

While it is perfectly possible to perform all of the above image processing steps, adding and removing features as necessary, in any image processing software such as Paint, for a large number of images to be processed it is preferential to automate this process as much as possible. In particular to remove annotations in the form of lines and marks is very helpful. To do this CurveClaw was developed to enable editing of images and selecting desired features / curves. Key steps embedded in CurveClaw are using a convex hull with four corner points to identify the graph area and cropping the image to this, subsequently transforming the shape onto a rectangle to ensure axes are straight. This is followed by the user inputting an integer *n*. The program analyzes pixel connectivity and sorts them into areas by this, with some associated size (pixel count), then all pixels in all areas but the *n* largest areas are set to be white. This effectively discards all small regions keeping only the *n* largest regions. The user can then specify the correct value of *n* to keep only desired areas and that image will be saved.

For selecting individual curves in more difficult cases, readily available curve extraction tools such as CurveSnap or WebPlotDigitizer were trialed but found to be insufficient. As such the CurveClaw includes its own curve selection tool. Here the image is displayed for user interaction and points can be selected by the user for a specific curve, the points selected are stored in that order. The nearest black pixel to each selected points is found, and if it is near the picture border it is also checked if there is a connected pixel on the border, these coordinates are then saved. When the curve is saved the coordinate list is used to construct a new curve in an empty image of equivalent size. Here if two sequential points are in the same region (determined using the labeled image as before) a minimum paths traversing only pixels belonging to that regions is found and plotted on an empty image, if the pixels are not in the same region a straight line is drawn between them instead. The minimum path is found by treating the image as a maze, where white pixels are walls and only black pixels with the correct label can be traversed, then the shortest path is simply the path between selected pixels with the fewest steps (only up, down, left and right steps are allowed). Sometimes more than one minimum distance path is found, due to the nature of the probabilistic data it is not necessary to find the perfect minimum paths and since only pixels on the original curve can be selected the error potentially incurred by this is likely negligible compared to the error in the data collection of the phase diagram as we always recover some part of the original curve. Once all curves have been defined, all the phase regions have then been extracted effectively. All curves are overlayed to reconstruct the phase diagram and the user gives information about the axis sizes and if log scales are present, this is used to construct the grid of test points and return the probabilistic data as described above. The workflow is summarized in Fig. 4, with blue boxes indicating steps which are readily automated and green boxes representing steps that require more manual input, not all steps are always necessary.

## Data Records

**Fig. 5:**
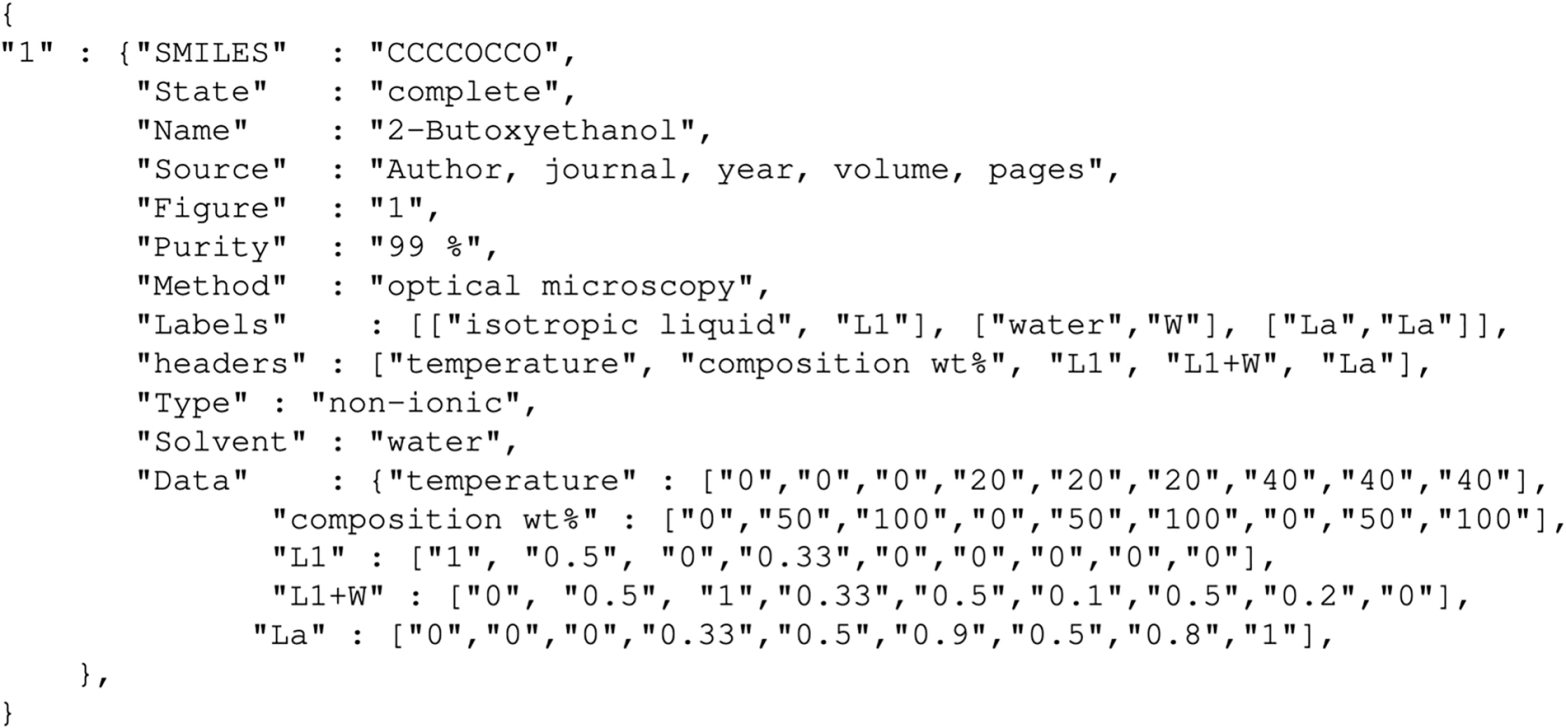
Example generic JSON record for a compound with SMILES string CCCCOCCO, with (hypothetical) phase states taken from Table 6.

The PhDat dataset containing all collected data is available under the CC BY 4.0 license and is hosted on figshare, accessible via the link 10.6084/m9.figshare.29071202^30^. Phase diagrams were processed using the methods described above and the results (the metadata and phase state probabilities for each phase diagram) were compiled into a JSON file structured as a list of records, indexed by a data record entry number. Each record thus contains data from one unique source, organized as a dictionary comprising: the SMILES string, the state of the diagram (either complete or incomplete if some areas are unknown), the name of the chemical compound, the source (e.g. the citation reference to the paper) and its figure location in the source (e.g. the figure number or page number), the purity of the chemical (if given), the measurement methodology in method, the type of the compound (nonionic, anionic, cationic, zwitterionic or mixed if both cation and anion are surfactants), the solvent which is water in all keys, the labels is a list of the original label assigned to a phase and the label this was assigned in the dataset, the keys for the data (header names) and then the values as a list for all data keys; the composition is always given as wt% (weight percent) of surfactant such that 0 wt% is pure solvent and 100 wt% is pure surfactant. Hence reading each column entry of the list of the set of data keys provides complete information on each discretized point of the diagram, e.g. its composition, temperature and the probability value (as a percentage) for each phase state. Each record retains the full temperature and composition grid corresponding to the original literature source. Note that this format allows for the same compound to have multiple records if there is more than one source for the phase diagram and one should *not* assume the SMILES strings are unique. To illustrate the record structure we show in Fig. 5 a generic example (record index “81”) for a compound with SMILES string CCCCOCCO and (hypothetical) phase states taken from Table 6, so that for instance at a temperature of 0 ^°^C and a composition of 50 wt% the probability that the state point is in the isotropic liquid *L*_1_ phase is 50%, the probability that it is in the cloud region (*L*_1_ + *W*) is also 50%, and the probability that it is a lamellar phase (*L*_*α*_) is 0% ; note that the grid size here does not reflect the grid size used in the actual dataset so as to better illustrate the changing probability as one moves across the diagram.

**Table 6:** Example output data: each row represents a particular temperature and composition point on the phase diagram for a specific molecule and gives the probability of that point being in a particular phase state.

temperature / ^°^C	composition / wt%	L1	L+W	La
0	0	1	0	0
0	50	0.5	0.5	0
0	100	0	1	0
20	0	0.33	0.33	0.33
20	50	0	0.5	0.5
20	100	0	0.1	0.9
40	0	0	0.5	0.5
40	50	0	0.2	0.8
40	100	0	0	1

## Technical Validation

In order to verify the automated data extraction a selection of Bell’s previously analyzed phase diagrams were digitized and sampled with the same grid size as in the original study^23^. Here only the phase state of that particular point was sampled using a simplified script compared to the one outlined above, which rather than assigning a probability gives each point a categorical assignment e.g. *L*_1_. The phase state assignments were then compared to Bell: only a handful of data points were found to have a different phase state assignment between our automated and Bell’s manual approach. All of these were grid points which fell onto or very near phase boundaries and as such could easily have been assigned as any of the adjacent phases by the manual approach. We note that the probabilistic assignment of phase states that we have adopted for the main dataset circumvents this problem in its entirety, since any point near a boundary would be assigned to all the nearby phases, with a vector of suitably weighted probabilities.

## Usage Notes

The data is provided as a JSON file, and once loaded, data for any of the given surfactants can be retrieved using the record index or be filtered by any of the keys such as the SMILES string. A link to an example workflow^31^ for retrieving data from the dataset is provided along with the dataset in the code availability section. A user may for example use a SMILES string to extract all data on that structure as a list of dictionaries, where each dictionary contains the data for one source. Similarly a user may iterate through all entries and extract only entries containing anionic surfactants. The JSON structure allows for many different ways to extract the desired data.

Initial analysis of the dataset indicates that there is an imbalance in observed phase states, as shown in Fig. 3, showing the number of data points and phase diagrams for each given phase. Some phase states are a dominant in many diagrams (e.g. *L*_1_, *L*_1_ + *W*), whereas other phase states such as *V*_1_ and *N*_1_ appear only as small regions in a handful of phase diagrams. Table 5 provides a complete list of phase states identified across all sources. The dataset contains normalized data across all phases for a given point on the phase diagram (for each surfactant and point in the phase diagram, the probability vector is normalized). For incomplete diagrams removing all points assigned a non-zero probability of U (unknown) phase state can be useful. Further since not all phase diagrams were measured across the same range of temperatures and compositions, it may be of use to only consider data points in a particular range.

We endeavored to adhere to the FAIR principles^32^, each entry in the database has a unique identifier, its index number, with each entry being given with descriptive keys, metadata, and sources. By using a JSON format we aim to make it universally accessible and easy to add to in future. With anyone being able to send us suggestions via 10.6084/m9.figshare.29071202^30^ (see Code Availability section) we plan to accumulate new data which will be regularly added to the dataset. We also plan to continually update PhDat with new data for different molecules and mixtures of molecules. By presenting data as reported in the original references while minimizing down stream processing we hope to stay accurate and allow for every user to make the processing decision themselves.

## Data Availability

The PhDat dataset is available under the CC BY 4.0 license and is hosted on figshare, accessible via the link 10.6084/m9.figshare.29071202^30^.

## Code availability

All Python scripts referred to in the article can be accessed via CurveClaw^29^, a bespoke program used for the semi-automated extraction of phase diagram data into digital (numerical) form. CurveClaw is available under BSD 2-clause license from https://github.com/RummelF/CurveClaw.git, a tutorial is provided along with the code. We also provide dataExplorer.py^31^, a Python-based command-line tool for exploring, analyzing, and summarizing phase-state data associated with chemical compounds or surfactants. It enables researchers to search compound databases using SMILES identifiers, extract phase probability data, and generate structured summaries for further analysis. This can be found at https://github.com/ashkurti/DataExplorer and is also available under a BSD 2-clause license.
